# Intermediate Filaments in Cellular Mechanoresponsiveness: Mediating Cytoskeletal Crosstalk From Membrane to Nucleus and Back

**DOI:** 10.3389/fcell.2022.882037

**Published:** 2022-04-11

**Authors:** Anne-Betty Ndiaye, Gijsje H. Koenderink, Michal Shemesh

**Affiliations:** Department of Bionanoscience, Kavli Institute of Nanoscience Delft, Delft University of Technology, Delft, Netherlands

**Keywords:** mechanobiology, migration, cytoskeleton, vimentin, keratin, actin, microtubules

## Abstract

The mammalian cytoskeleton forms a mechanical continuum that spans across the cell, connecting the cell surface to the nucleus *via* transmembrane protein complexes in the plasma and nuclear membranes. It transmits extracellular forces to the cell interior, providing mechanical cues that influence cellular decisions, but also actively generates intracellular forces, enabling the cell to probe and remodel its tissue microenvironment. Cells adapt their gene expression profile and morphology to external cues provided by the matrix and adjacent cells as well as to cell-intrinsic changes in cytoplasmic and nuclear volume. The cytoskeleton is a complex filamentous network of three interpenetrating structural proteins: actin, microtubules, and intermediate filaments. Traditionally the actin cytoskeleton is considered the main contributor to mechanosensitivity. This view is now shifting owing to the mounting evidence that the three cytoskeletal filaments have interdependent functions due to cytoskeletal crosstalk, with intermediate filaments taking a central role. In this Mini Review we discuss how cytoskeletal crosstalk confers mechanosensitivity to cells and tissues, with a particular focus on the role of intermediate filaments. We propose a view of the cytoskeleton as a composite structure, in which cytoskeletal crosstalk regulates the local stability and organization of all three filament families at the sub-cellular scale, cytoskeletal mechanics at the cellular scale, and cell adaptation to external cues at the tissue scale.

## Introduction

The cytoskeleton is a fascinating cellular machinery that performs multiple, to some extent contradictory, functions. It acts as a stable structural scaffold providing cells with a specific functional shape and protecting against external forces. Accordingly, genetic defects in cytoskeletal proteins are associated with mechanical defects in cells and tissues, which for instance result in kidney scarring (Feng et al., 2018), skin fragility (Haines and Lane 2012), and muscle failure (Adil et al., 2021). On the other hand, the cytoskeletal structures are also dynamic enough to enable cell migration, division and mechanosensitive response to the environment (Chaudhuri et al., 2020).

**FIGURE 1:**
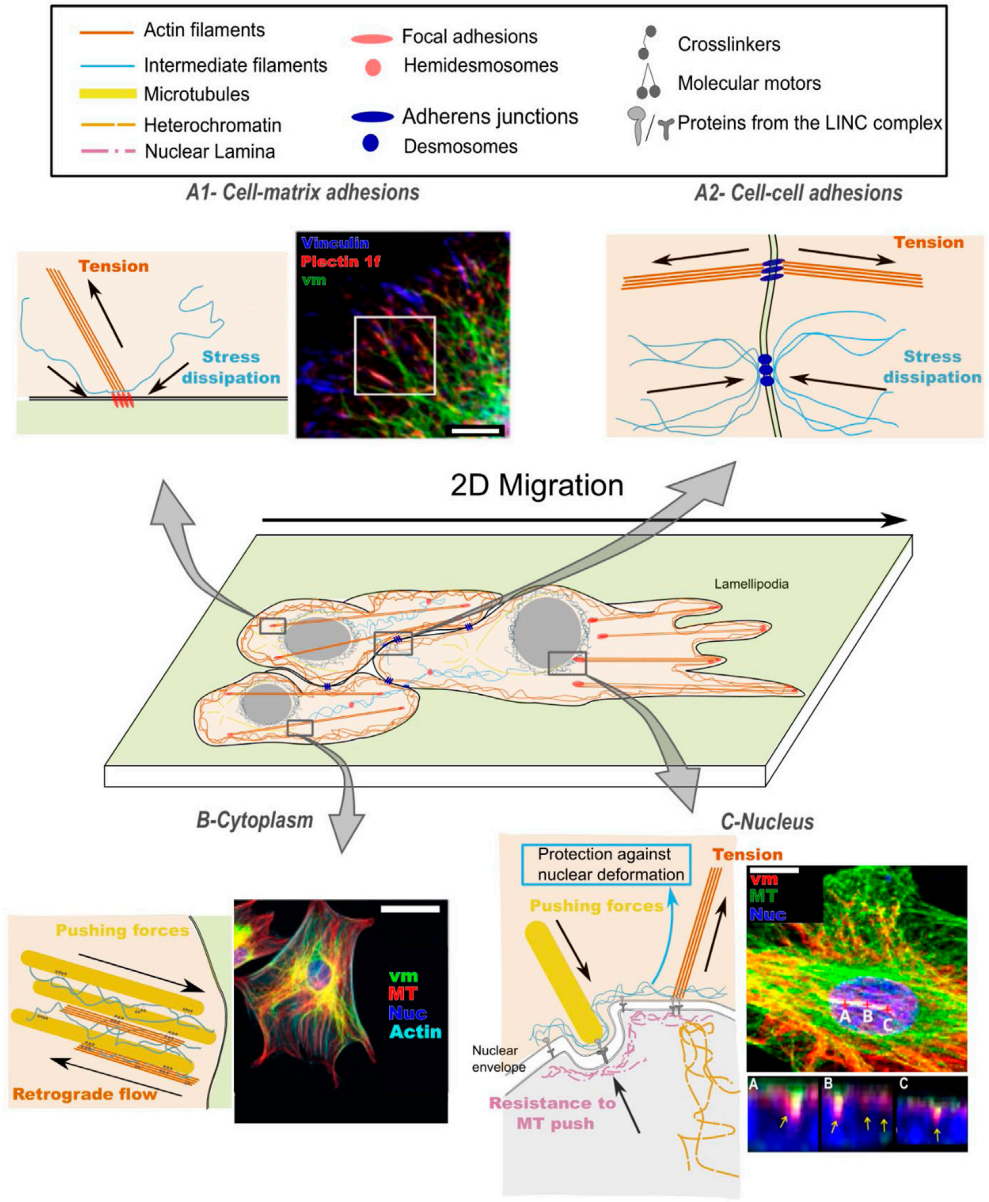
Cytoskeletal crosstalk between intermediate filaments (IFs), actin filaments (AFs) and microtubules (MTs) contributes to mechanosensing: **(A)** at the cell surface: mechanical signals emerging from the matrix **(A1)** or from neighboring cells **(A2)**; **(B)** in the propagation of mechanical signals across the cell cytoplasm and **(C)** up to the cell nucleus. **(A1)** At cell-matrix adhesions, actin/vimentin crosstalk regulates focal adhesion turnover, which results in dissipation of local stresses. Fluorescence image from (Gregor et al., 2014) shows the intimate spatial relation between vimentin (vm), plectin and vinculin, orchestrating focal adhesion turnover; scale bar is 10 µm. **(A2)** At cell-cell adhesions, actin/intermediate filament crosstalk is activated upon tensile (pulling) forces and participates in the regulation of cellular prestress. **(B)** In the dense cytoplasm, mechanical forces are transmitted through all three cytoskeletal networks. The intermediate filament network affects the (de)polymerization rates of the other two networks, and the three networks co-align. Fluorescence image from (Vohnoutka et al., 2019) demonstrates the dense organization of vimentin, microtubules (MT) and actin. Vimentin spans from membrane sites to the nucleus (DAPI) and forms a cage surrounding the nucleus. Scale bar is 50 µm. **(C)** At the nucleus, forces are transmitted between the cytoskeleton and chromatin through LINC complexes, affecting nuclear shape and heterochromatin density, while intermediate filaments protect the nucleus from large deformations. Fluorescence image from (Feliksiak et al., 2020) shows microtubules and vimentin around the nucleus (DAPI); scale bar = 15 µm. Areas marked by A-B-C demonstrate the tight association of vimentin with nuclear grooves.

Although the cytoskeleton is highly dynamic at the subcellular (nm) scale, it nevertheless maintains structural integrity at the cell scale (microns) and at the tissue scale (up to millimeters). This disparity is most likely due to the composite nature of the cytoskeleton, based around three protein filament networks with distinct structural, mechanical and biochemical properties: actin filaments, microtubules, and intermediate filaments (Figures 1A,B). All three filaments are reversible polymers that self-assemble from weakly interacting subunits whose local availability is a critical determinant of local cytoskeletal dynamics (Plastino and Blanchoin 2018; Ohi et al., 2021). Both actin filaments and microtubules are structurally polar filaments, respectively composed of actin monomers that hydrolyze ATP, and tubulin dimers that hydrolyze GTP. They both exhibit fast ( ∼ seconds to minutes) turnover rates fueled by ATP/GTP hydrolysis (Theriot and Mitchison 1991; McNally 1996). By contrast, intermediate filaments are non-polar filaments that lack intrinsic enzymatic activity (Li et al., 2006; Robert et al., 2015). Their remodeling occurs by slow ( ∼ hours) exchange of filamentous tetramers (Çolakoğlu and Brown 2009; Nöding et al., 2014; Robert et al., 2015). It has been proposed that this long-lived intermediate filament network mechanically integrates the cytoskeleton and provides structural memory that helps maintain cell polarity (Gan et al., 2016).

Each of the three cytoskeletal networks has its own set of dedicated regulatory proteins that control their structure, dynamics and mechanics with high spatial and temporal precision (Plastino and Blanchoin 2018; Dutour-Provenzano and Etienne-Manneville 2021; Gudimchuk and McIntosh 2021). Cells are thus able to form different specialized cytoskeletal arrays, such as the branched actin networks at the leading edge of migrating cells, the microtubule spindle in dividing cells, and an intermediate filament cage-like structure that protects the nucleus of cells during confined migration. The posttranslational addition of various chemical groups further enhances the complexity of the cytoskeletal proteome, hence the ability of the cell to fine-tune cytoskeletal functions (MacTaggart and Kashina 2021).

It is increasingly recognized that the functions of the three structural systems are tightly coupled *via* crosslinkers, motors, adhesion complexes and shared signaling factors. Recently, our understanding of the molecular mechanisms of cytoskeletal crosstalk and its consequences for cell shape, mechanics and fundamental processes such as directional migration, has grown (Huber et al., 2015; van Bodegraven and Etienne-Manneville 2020). Still, the role of cytoskeletal crosstalk in cellular mechanosensitivity remains poorly understood. Traditionally actin filaments are considered as the main contributor to mechanosensitivity, since they actively apply contractile (traction) forces to the extracellular matrix and to adjacent cells *via* specialized adhesion complexes and transmit mechanical signals to the nucleus, for instance *via* LINC complexes (Sun et al., 2016; Yap et al., 2018). Microtubules are thought to play mainly a regulatory role through their interactions with the actin cytoskeleton and with cell-matrix and cell-cell adhesions (Dogterom and Koenderink 2019; Rafiq et al., 2019; Pimm and Henty-Ridilla 2021). Intermediate filaments are usually considered as passive cytoskeletal elements that maintain cell/tissue integrity. However, there is a growing appreciation that intermediate filaments play a central role in cellular mechanosensitivity, forming a mechanically strong yet responsive network that links to the actin and microtubule cytoskeleton, cell adhesions, and nuclear complexes (Chang and Goldman 2004). This is especially intriguing because intermediate filaments exhibit much more tissue diversity than the other cytoskeletal subsystems. The actin and microtubule cytoskeleton are differently organized in different cell types, but their molecular composition is relatively conserved, with a limited number of isoforms. By contrast, there are ∼ 70 intermediate filament genes in humans, with further diversity arising from alternative splicing. For instance, different types of epithelial cells express different sets of keratins, mesenchymal cells express vimentin, muscle cells express desmin, and neurons express neurofilaments. Intermediate filaments are hence widely used as markers for differentiation (Redmond and Coulombe 2021; Sjöqvist et al., 2021). Herein, we review evidence pointing to the importance of cytoskeletal crosstalk in cellular mechanosensitivity, with a particular focus on the role of intermediate filaments. We demonstrate how intermediate filaments span from cell-cell and cell-matrix adhesion sites, through the cytoplasm and up to the nucleus (see Figure 1), thereby orchestrating long-range mechano-chemical crosstalk between the cytoskeleton, cell adhesions, and internal processes.

## Contributions of Cytoskeletal Crosstalk to Mechanosensing at the Cell Membrane

### Mechanosensing at Cell/Extracellular Matrix Contacts

Cell-matrix contacts are key players in mechanotransduction as they enable cells to apply forces on the extracellular matrix, in response to its mechanical properties (Sun et al., 2016). These matrix contacts are essential for determining cell polarity, directional migration and the cell’s ability to remodel the extracellular matrix (Brabletz et al., 2021). The role of actin-based transmembrane adhesion complexes, including but not limited to focal adhesions, in mechanosensitivity has been extensively reviewed (Kechagia et al., 2019). The role of the other two cytoskeletal filament families in mechanosensitivity of cell-matrix contacts is recently gaining more attention (Leube et al., 2015; Sanghvi-Shah and Weber 2017; van Bodegraven and Etienne-Manneville 2020).

Focal adhesions are mostly identified with actin-based structures; however, multiple intermediate filament proteins have also been identified at focal adhesions (Figure 1A1). In epithelial cells, keratin filaments are nucleated at focal adhesions and transported inwards assisted by the actin cytoskeleton (Windoffer et al., 2006; Leube et al., 2015). In fibroblasts and endothelial cells, vimentin filaments are anchored to focal adhesions and regulate their size and adhesion strength (Bhattacharya et al., 2009; Kim et al., 2010; Gregor et al., 2014; Kim et al., 2016; Terriac et al., 2017; Vohnoutka et al., 2019). This involves direct force transmission from cell surface integrins to the cell interior (Maniotis et al., 1997), regulating actin stress fibers (Jiu et al., 2015; Jiu et al., 2017) and the associated mechanosensing machinery (Gregor et al., 2014). Focal adhesion anchoring enables vimentin to promote integrin-mediated activation of the major mechanosensor focal adhesion kinase (FAK) and its downstream tension-dependent signaling cascade (Gregor et al., 2014). Actomyosin-dependent rigidity sensing controls microtubule acetylation, which in turn tunes the mechanosensitivity of focal adhesions (Seetharaman et al., 2021). Since microtubule acetylation also affects the association of intermediate filaments with actin bundles at focal adhesions, this points to a complex three-way feedback mechanism that still remains to be disentangled. Cell adhesion is tightly connected to migration; Vimentin tunes cell migration through collagen and fibronectin matrices (Ding et al., 2020; Ostrowska-Podhorodecka et al., 2021, Ostrowska-Podhorodecka, Ding et al. 2021). Persistent collective migration of astrocytes was dependent on the size and turnover of focal adhesions, in a cell-specific (leader *vs*. follower) manner. The regulation of focal adhesions and cell-cell contacts requires the intermediate filament network, which is composed mainly of glial fibrillar acidic protein (GFAP), vimentin, and nestin (Moeton et al., 2016; De Pascalis et al., 2018). Intermediate filaments also modulate traction forces; in migrating fibroblasts, the vimentin cytoskeleton was shown to slow down actin retrograde flows, while promoting orientation of actin stress fibers and traction forces (Costigliola et al., 2017). Intermediate filaments modulate also forces oriented perpendicularly to the substrate, through invadopodia (Schoumacher et al., 2010), possibly *via* interacting with actin capping proteins (Lanier et al., 2015).

Intermediate filaments are further anchored to the cell surface by proteins of the plakin family, specifically plectin in hemidesmosomes and desmoplakin in desmosomes (Dowling et al., 1996; Mohammed et al., 2020). Plectin is a major crosslinker connecting the three cytoskeletal filament families (Svitkina et al., 1996; Wiche et al., 2015). Molecular dynamic simulations suggested that plakins act as mechanosensors: pulling forces resulted in plectin and desmoplakin unfolding and exposure of the SH3 domain, which may potentially trigger downstream signaling cascades (Daday et al., 2017). Experimentally, activated plectins were shown to promote microtubule destabilization through their interaction with MAP2, which antagonizes the MAP2-mediated stabilization of MTs (Valencia et al., 2013). Recent studies indicate that FAs and hemidesmosomes are mechanically coupled (Nardone et al., 2017; Wang et al., 2020).

### Mechanosignalling at Cell-Cell Contacts

Cell-cell interactions play a crucial role in physiological mechanosensitive processes such as tissue morphogenesis, but also in pathological processes such as inflammatory bowel diseases (Adil et al., 2021). Cells interact through cadherin-based adherens junctions that connect the actin networks of neighboring cells in epithelia and endothelia, and desmosomes that connect the intermediate filaments of neighboring cells and reinforce tissues that experience high mechanical stress such as the epidermis (Rübsam et al., 2018; Broussard et al., 2020) (Figure 1A2). Besides providing mechanical coherence, both adhesions are involved in cell and tissue adaptation to mechanical cues (Charras and Yap 2018; Angulo-Urarte et al., 2020; Zuidema et al., 2020). The mechanosensitivity of adherens junctions was shown to rely on force-sensitive conformational changes of *α*-catenin and vinculin (Yao et al., 2014; Seddiki et al., 2018). Desmosomes are sites of local assembly/reorganization of keratin filaments (Kim et al., 2021), similar to hemidesmosomes. Desmoplakin, one of the core proteins of desmosomes that binds keratin filaments (Bornslaeger et al., 1996), was recently shown to experiences forces in the pN range in stretched epithelial monolayers, suggesting its load-bearing function (Price et al., 2018).

Although previous studies examining adherens junctions (Engl et al., 2014) and desmosomes (Price et al., 2018) considered the different cytoskeletal networks separately, there is evidence that the three cytoskeletal elements interdependently modulate the dynamical properties of cell-cell junctions. Microtubules promote actin recruitment at adherens junctions and intercellular transmission of the contractile forces generated by the actomyosin network (Ko et al., 2019). In migrating astrocytes, intermediate filaments influence actin-driven retrograde flow of adherens junctions (De Pascalis et al., 2018). In migrating epithelial cells, desmosome dynamics was shown to depend on both intermediate filaments and actin (Roberts et al., 2011). Intermediate filaments also appear to be involved in vascular permeability (Bayir and Sendemir 2021) by helping to organize continuous adherens junctions and the underlying actin network *via* plectin crosslinking (Osmanagic-Myers et al., 2015). In epithelia, plectin mechanically couples cortical keratin and actin networks and ensures a uniform distribution of actomyosin-generated forces (Prechova et al., 2022). Finally, growing evidence points to collaboration between intermediate filament-desmosome and actin-adherens junction networks during mechanosensing and force generation (reviewed in (Zuidema et al., 2020)).

## Contributions of Cytoskeletal Crosstalk to Force Transmission Through the Cytoplasm

Physical interactions between intermediate filaments, actin, and microtubules influence the mechanical properties of the cytoskeleton as a whole, and hence force transmission from the cell surface to the nucleus (Figure 1B). The three cytoskeletal filaments strongly differ in their bending rigidity, as quantified by the persistence length, *l*_p_. Intermediate filaments are most flexible, with *l*_p_ ≈ 0.5–2 μm, microtubules are most rigid, with *l*_p_ ≈ 1–10 mm, and actin filaments are intermediate with *l*_p_ ≈ 8 μm (Huber et al., 2015). The filaments also strongly differ in their rupture strain: actin filaments and microtubules only support small tensile strains whereas intermediate filaments support large elongations because their subunits can slide and unfold (Block et al., 2017). Understanding how these single-filament properties translate in cell-scale mechanics is challenging given the molecular and structural complexity of the cytoskeleton. Cell-free reconstitution experiments are hence essential to elucidate the individual and collaborative roles of the different cytoskeletal filaments in cytoskeletal mechanics.

Reconstituted networks of purified actin and intermediate filaments (vimentin or keratin) strain-stiffen when exposed to shear or tensile stresses. These filaments are semiflexible, with a persistence length that is of the same order as the contour length. Experiments and theoretical modelling demonstrated that strain-stiffening occurs because the thermally undulating filaments are straightened out by tensile strains, which reduces the conformational entropy of the fluctuating polymer segments between adjacent crosslinks, and hence opposes further deformation (Gardel et al., 2004; Broedersz and MacKintosh 2014). Depending on the time scale of the imposed mechanical load, reconstituted vimentin networks can additionally dissipate mechanical stress because crosslinks between filaments can remodel and the filaments themselves lengthen by subunit unfolding and sliding elongations (Aufderhorst-Roberts and Koenderink 2019; Forsting et al., 2019). Combining the different cytoskeletal polymers in composite networks demonstrates intriguing co-dependent mechanical properties. Actin/vimentin and actin/microtubule mixtures were shown to exhibit enhanced stiffness and compressibility compared to the single-component networks (Esue et al., 2006; Pelletier et al., 2009; Lin et al., 2011). Furthermore, microtubules were shown to counteract myosin motor-driven contraction of actin networks through their ability to bear large compressive loads (Lee et al., 2021a). Physical interactions also introduce co-dependent polymerization dynamics of the three cytoskeletal polymers. Branched actin networks reduce the growth rate of microtubules and trigger their depolymerization (Colin et al., 2018), while vimentin filaments bind to microtubules and stabilize them against depolymerization (Schaedel et al., 2021) and also bind to actin filaments (Esue et al., 2006). In the presence of crosslinkers and motors, the three filament systems can additionally co-align and (re-)direct each other’s polymerization direction (Preciado López et al., 2014; Gan et al., 2016; Leduc and Etienne-Manneville 2017).

These physical effects identified in simplified reconstituted systems likely contribute to mechanical co-dependencies observed in cells, such as toughening by stress dissipation in the vimentin network (Hu et al., 2019), protection against compressive forces by the vimentin network (Mendez et al., 2014), and vimentin-dependent modulation of actin-myosin contractility (De Pascalis et al., 2018). Raman imaging recently showed that actomyosin forces are transmitted to the intermediate filament cytoskeleton: cells on rigid substrates, where myosin contractility is high (Gupta et al., 2019), contained more unfolded vimentin than on soft substrates, where tension is low (Fleissner et al., 2020). In epithelial monolayers a similar mechanical interplay between the actin and intermediate filament networks was found (Latorre et al., 2018), where cell stretching dilutes the actin cortex and hence decreases tension, while keratin filaments that bear tension re-stiffen the cells. There is evidence that microtubules also contribute to the overall mechanical balance; epithelial folding was for instance shown to emerge from the balance between myosin contractile forces and microtubule-generated pushing forces (Takeda et al., 2018). It would be interesting to evaluate more systematically how mechanical co-dependencies among the three cytoskeletal filament families respond to modified substrate stiffness.

## Contributions of Cytoskeletal Crosstalk to Mechanosensitivity at the Nucleus

The nucleus plays a key role in mechanotransduction and mechanosensing (reviewed in (Kirby and Lammerding 2018; Janota et al., 2020)). Nuclear chromatin and the cytoskeleton are physically linked through the LINC (Linker of Nucleoskeleton and Cytoskeleton) complex (Bouzid et al., 2019), which is associated with chromatin and nuclear lamins, members of the intermediate filament family (Figure 1C). Actin filaments are anchored to the nucleus *via* nesprin-1 and nesprin-2, intermediate filaments *via* nesprin-3, and microtubules *via* nesprin-4 (Zhen et al., 2002; Warren et al., 2005; Wilhelmsen et al., 2005; Roux et al., 2009). Considering the role of intermediate filaments in mechanical stabilization of the nucleus, as shown for vimentin (Patteson et al., 2019) and keratin (Almeida et al., 2015), we propose that future work should focus further on resolving the interactions between the three cytoskeletal components at the nuclear envelope in response to changes in substrate rigidity, for instance by superresolution microscopy and molecular tension sensors (Arsenovic et al., 2016; Leduc and Etienne-Manneville 2017).

The physical links between the nuclear lamins and the cytoskeleton provide continuous feedback between the mechanical properties of the nucleus of the cell and its environment (Buxboim et al., 2014; Lomakin et al., 2020; Venturini et al., 2020). Soft substrates promote phosphorylation and turnover of lamin A/C, resulting in softer and less spread nuclei (Buxboim et al., 2014). Pushing forces on the nuclear envelope exerted by microtubules are balanced by the laminA network for the maintenance of a round nuclear shape (Ramdas and Shivashankar 2015; Tariq et al., 2017). In differentiating Hematopoietic Stem and Progenitor (HSPC) cells, local nuclear invaginations associated with centrosomes and microtubule bundles depend on the laminB density and the activity of dynein (Biedzinski et al., 2020). Such local interactions at the nucleus possibly depend on environmental cues. In MEFs plated on micropatterned substrates with independent control over the overall cell shape and the focal adhesion size, the cell-ECM contact size was shown to have more impact than cell shape on overall cell polarization, in a LaminA dependent manner (Lee et al., 2021b). These results strengthen the notion that cytoskeletal crosstalk affects mechanoresponsiveness all the way from the cell surface to the nucleus.

## Discussion

In this mini-review, we gathered recent evidence demonstrating the contribution of cytoskeletal crosstalk in transferring mechanical signals from contact points at the plasma membrane to the nucleus. Intermediate filaments play a central role in this crosstalk, by interacting with the actin and microtubule cytoskeleton, cell-cell and cell-matrix adhesions, and nuclear complexes. We propose to shift focus in cytoskeletal and mechanobiological research towards a more holistic view of the cytoskeleton as a composite structure, examining the responses of *all* three structural families to mechanical cues. The central role of intermediate filaments in mechanosensitivity may render cell/tissue-specific mechanosensitivity. Moreover, during development, aging or pathology, the composition of the intermediate filament cytoskeleton undergoes major changes (Redmond and Coulombe 2021; Sjöqvist et al., 2021). This raises the hypothesis that there may be an “intermediate filament code” that confers cell type-specific mechanosensitive functions.

Elucidating the mechanisms by which intermediate filaments contribute to mechanosensing and mechanotransduction is far from trivial given the molecular complexity of the cytoskeletal proteome together with its cell/tissue specificity. Connecting the manifold molecular-scale interactions to the emergent mechano-biological functions at the cellular level is also challenging. To delineate the functions of different intermediate filament proteins across scales, we believe that it is vital to combine studies in cell culture models and model organisms, where cells can be studied in their native context, with studies of “clean” reconstituted systems, where cytoskeletal crosstalk can be studied under controlled conditions to facilitate combinations with predictive models.

**TABLE 1:** Selected examples of known cytoskeletal crosstalk interactions relevant for environmental mechanosensing that involve intermediate filaments. Interactions are sorted by subcellular localization, noting the structural and crosslinker proteins known to be involved in the crosstalk, as well as the major cellular function.

Localization	Relevant Cytoskeletal Filaments	Interacting Proteins	Cellular Function	References
Ventral membrane (Focal adhesions; epithelial cells)	Keratin	Zyxin	Focal adhesions control keratin formation, turnover and transport	(Windoffer et al., 2006; Leube et al., 2015)
		Paxillin		
	Actin	Talin		
Ventral membrane (Focal adhesions; mesenchymal cells)	Vimentin	Plectin	Vimentin restricts focal adhesion size and regulates integrin trafficking; focal adhesions control vimentin organization	(Bhattacharya et al., 2009; Kim et al., 2010; Gregor et al., 2014; Kim et al., 2016; Terriac et al., 2017; Vohnoutka et al., 2019)
		Integrins β1, β3		
		Vinculin		
	Actin	FAK		
		Hic-5		
		Filamin A		
Lamellipodia (Fibroblasts)	Vimentin	RAC1	Vimentin detachment from membrane sites is essential for lamellipodia formation	Helfand et al. (2011)
	Actin			
Membrane: (hemidesmosomes; epithelial cells)	Keratin	Integrin α6β4	Hemidesmosomes control keratin organization, likely important for tissue resilience	(Colburn and Jones 2018; Moch and Leube 2021)
	Actin			
	Microtubules			
Membrane: (Cell-Cell junctions + leading edge; astrocytes)	Vimentin	Paxillin	Vimentin promotes collective directed migration by regulating actomyosin traction force generation	De Pascalis et al. (2018)
		Plectin		
	Actin	N-Cadherin		
		E-Catenin		
Cortex	Vimentin	Plectin	Vimentin interaction maintains cortex tension, required for cell division of confined cells	(Duarte et al., 2019; Serres et al., 2020)
	Actin			
Cytoplasm (mesenchymal cells)	Vimentin	Plectin	Plectins crosslink the cytoskeletal networks for cell integrity; vimentin regulates actin stress fibers	(Svitkina et al., 1996; Jiu et al., 2017)
	Actin			
	Microtubules			
Cytoplasm (mesenchymal cells)	Vimentin	Plectin	Actin arcs drive perinuclear vimentin accumulation; vimentin restrains width of the actin-filled lamellum	(Jiu et al., 2015; Lowery et al., 2015)
	Actin			
Cytoplasm	Actin	Plectin	Matrix rigidity sensing and cell mechanical properties	(Guo et al., 2013; Bonakdar et al., 2015; Laly et al., 2021)
	Keratin14			
	Lamin A/C	Paxillin		
Nucleus	Vimentin	LINC complex formed by sun and nesprin proteins	Nucleo-cytoskeletal force transmission maintains nuclear position under strain and during migration	(Folker et al., 2011; Lombardi et al., 2011; Marks and Petrie 2022)
	Actin			
	Microtubules			
	Lamin A/C			
